# A Case of Takotsubo Cardiomyopathy After Local Anesthetic and Epinephrine Infiltration

**DOI:** 10.7759/cureus.3173

**Published:** 2018-08-21

**Authors:** Assad Ali, Azfar K Niazi, Paul Minko, Partha J Saha, Kavita Elliott, Nikhil Bhatnagar, Sabry Ayad

**Affiliations:** 1 Anesthesia, Cleveland Clinic Foundation, Cleveland , USA; 2 Outcomes Research Department, Cleveland Clinic, Cleveland, USA; 3 Anesthesia Institute, Cleveland Clinic Foundation - Fairview Hospital, Cleveland, USA

**Keywords:** takotsubo cardiomyopathy, stress cardiomyopathy, local anesthetic toxicity, heart failure, gynecological surgery, female urology, critical care

## Abstract

Takotsubo cardiomyopathy (TC) is a non-ischemic cardiomyopathy that is accompanied by sudden left ventricular myocardial stunning, dilation and dysfunction. It often results from severe emotional or physical stress. We present the case of a 41-year-old female patient who had general anesthesia induced uneventfully for an elective bladder sling procedure. After an intravaginal injection of local anesthesia (lidocaine 2%; epinephrine 1:100,000) just prior to the surgical incision, the patient had cardiovascular collapse for which cardiopulmonary resuscitation (CPR) was performed. The patient was eventually stabilized but transesophageal echocardiography showed impairment in cardiac motion and remarkably reduced ejection fraction. Troponin levels were elevated but coronary angiography was unremarkable. The ejection fraction returned to normal the next day. Local anesthetic with epinephrine administration can lead to TC, and with optimal management, long-term cardiac sequela can be avoided.

## Introduction

Takotsubo cardiomyopathy (TC) is a rare heart condition characterized by left ventricular apical ballooning leading to acute cardiac dysfunction without the presence of atherosclerotic plaques in the coronary arteries [1]. Historically, TC has also been referred to as ‘broken heart syndrome’ due to its increased incidence in post-menopausal women undergoing acute physical or emotional stress [2]. TC was initially described by Japanese cardiologists in the early 1990s after echocardiographic imaging revealed images where the left ventricle was severely dilated resembling an octopus pot [3]. Since the initial description, several mechanisms have been proposed for the pathogenesis of TC. A mechanism that has gained support over the past few years traces TC to an abnormal reaction to catecholamines released during the body’s stress response [3-6]. In this report, we discuss a case of TC occurring after a patient received a surgical site local infiltration of local anesthetic mixed with epinephrine.

## Case presentation

**Figure 1:**
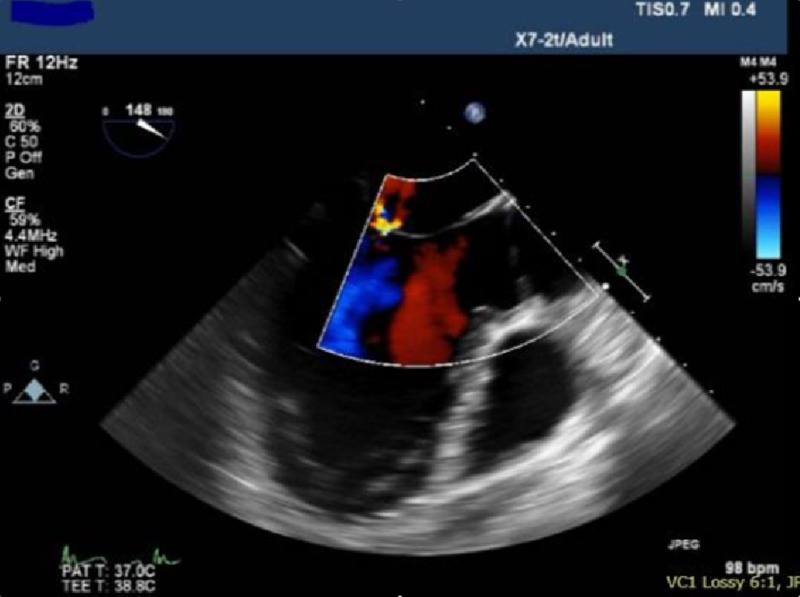
Transesophageal echocardiography done in the operating room (after the patient collapsed) showing systolic dysfunction of the left ventricle

A 41-year-old obese female with a medical history of depression, post-traumatic stress disorder and premature ventricular contractions (PVC) on propranolol, sertraline, alprazolam and phenazopyridine, was admitted for elective bladder sling procedure. The patient had a normal preoperative evaluation and pre-anesthesia consultation. Laboratory values were unremarkable. A recent echocardiogram revealed an ejection fraction of 55%-60%. General anesthesia was induced uneventfully with midazolam, propofol, rocuronium, lidocaine and fentanyl. Prior to starting the surgical procedure and thirty minutes after induction, an intravaginal injection of 10 mL of lidocaine 2% with epinephrine 1:100,000 for local anesthesia was administered. Within a minute after local anesthetic administration, the patient developed severe bradycardia. Despite administration of epinephrine and atropine, the patient’s condition worsened to pulseless electrical activity. Chest compressions were started along with supportive therapy using vasopressors and inotropic agents and the patient subsequently developed pulmonary edema. A dose of 100 mL of intralipid (a chelating agent for lidocaine) for suspected local anesthetic toxicity was given to the patient. Supportive therapy with milrinone, epinephrine and norepinephrine were used which eventually stabilized the patient. An intraoperative transesophageal echocardiography (TEE) showed global impairment in cardiac motion—septal dyskinesia; left ventricular hypokinesia, severe dilation with systolic dysfunction; anterior wall abnormalities without right ventricular strain and an ejection fraction of 20% as shown in the figure below (Figure 1). An electrocardiogram revealed prolonged QT and non-specific t-wave abnormalities.

**Figure 2:**
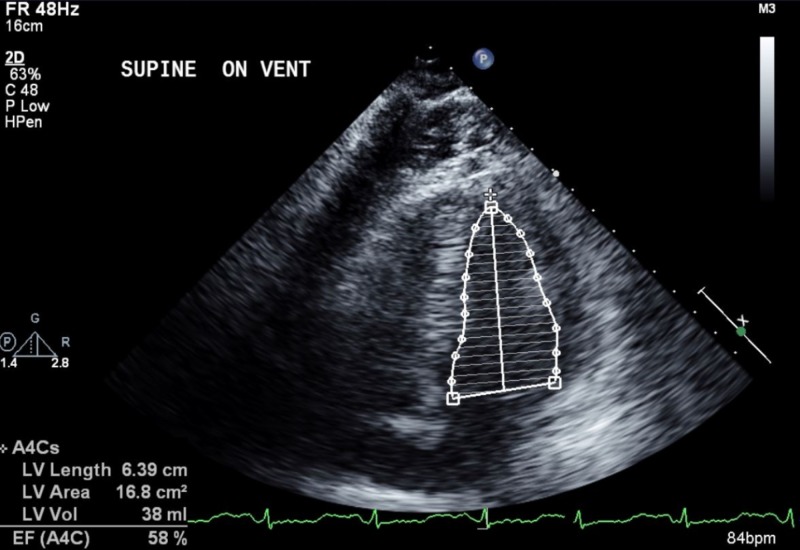
Transthoracic echocardiogram of the left ventricle showing normalization of the ejection fraction less than 36 hours after cardiovascular collapse

Pulmonary embolism was ruled out. Due to the suspected acute coronary syndrome, the patient was taken to the cardiac catheterization lab, where an intra-aortic balloon pump was placed to increase cardiac output and decrease afterload. Troponin T values were found to be elevated (1.03 ng/mL) and coronary angiography showed no evidence of coronary artery obstruction/plaque rupture leading to the diagnosis of TC. The patient was transferred to the intensive care unit where supportive therapy was continued, and the next day, a follow-up echocardiography showed an improvement of ejection fraction to 58% (Figure 2).

## Discussion

Several mechanisms have been suggested for the development of TC. A strong proposal for the pathogenesis of TC is that it arises as a result of an abnormal response to catecholamines released by the sympathetic nervous system during the body’s stress response [7]. More often than not, this phenomena has been observed in post-menopausal women who have recently undergone an acute emotional stress. The result of this stress is a hyper-activation and subsequent hyper-reaction to epinephrine and norepinephrine released during an acute emotional incident. In a small subset of patients, TC can be triggered by physical stressors [8]. Similar to the body’s physiologic response to an acute emotional stress, the outpouring of catecholamines during a cardiac arrest can also trigger TC [4]. It is important to note that TC patients who progress to cardiac arrest during surgery and receive inotropes (i.e., epinephrine and norepinephrine) will be exposed to further exacerbation of their condition from excessive doses of exogenous catecholamine used during resuscitation. Therefore, before coronary angiography can be arranged, a speckle-tracking-derived echocardiography (STE) can help in identifying the acute phase of TC. In such patients, catecholamines should be used only if absolutely necessary [9].

It has been stated in the literature that the myocardial stunning is often observed on echocardiography for patients diagnosed with Takotsubo. Epinephrine activates β-adrenoreceptors which are found in high density at parts of the apical myocardium, suggesting, that an abnormal response to a high flux of epinephrine after an acute stress can result in negative inotropic leading to systolic dysfunction [5].

## Conclusions

We have presented the case of a middle-aged female patient with no significant cardiac history other than PVC’s on propranolol, who was scheduled to undergo an elective bladder sling procedure. Anesthesia was induced uneventfully; however, after local infiltration of lidocaine mixed with epinephrine, the patient went into pulseless electrical activity. The patient was ressucitated and maintained on supportive therapy. TEE revealed cardiac compromise with a markedly depressed ejection fraction. Cardiac catherization ruled out acute coronary syndrome. Absence of coronary artery occlusion resulted in TC diagnosis. Our patient was managed using supportive therapy similar to that of patients suffering from acute heart failure (i.e., inotropes and vasopressors). What we can ascertain from this case is that TC is a diagnosis of exclusion in the setting of acute cardiac dysfunction without the presence of plaques or evidence of plaque rupture in coronary arteries.

## References

[1] Akashi YJ, Goldstein DS, Barbaro G, Ueyama T (2008). Takotsubo cardiomyopathy: a new form of acute, reversible heart failure. Circulation.

[2] Takotsubo Cardiomyopathy (2018). Takotsubo cardiomyopathy: background, pathophysiology, etiology. https://emedicine.medscape.com/article/1513631-overview.

[3] Komamura K, Fukui M, Iwasaku T, Hirotani S, Masuyama T (2014). Takotsubo cardiomyopathy: pathophysiology, diagnosis and treatment. World J Cardiol.

[4] Madias JE (2016). Cardiac arrest-triggered takotsubo syndrome vs. takotsubo syndrome complicated by cardiac arrest. Int J Cardiol.

[5] Mori H, Ishikawa S, Kojima S (1993). Increased responsiveness of left ventricular apical myocardium to adrenergic stimuli. Cardiovasc Res.

[6] Wittstein IS (2012). Stress cardiomyopathy: a syndrome of catecholamine-mediated myocardial stunning?. Cell Mol Neurobiol.

[7] Ansari U, El-Battrawy I, Fastner C (2018). Clinical outcomes associated with catecholamine use in patients diagnosed with Takotsubo cardiomyopathy. BMC Cardiovasc Disord.

[8] Hessel EA, London MJ (2010). Takotsubo (stress) cardiomyopathy and the anesthesiologist: enough case reports. Let’s try to answer some specific questions!. Anesth Analg.

[9] Daniel M, Hetzer R (2018). Deleterious effects of catecholamine administration in acute heart failure caused by unrecognized Takotsubo cardiomyopathy. BMC Cardiovasc Disord.

